# Exploration of the acceptability and usability of advance care planning tools in long term care homes

**DOI:** 10.1186/s12904-020-00689-9

**Published:** 2020-12-22

**Authors:** Tamara Sussman, Sharon Kaasalainen, Rennie Bimman, Harveer Punia, Nathaniel Edsell, Jess Sussman

**Affiliations:** 1 McGill University School of Social Work, 3506 Rue University #300, Montréal, QC H3A 2A7 Canada; 2 Health Sciences Centre, McMaster University School of Nursing, 2J20, 1280 Main Street West, Hamilton, ON L8S 4K1 Canada; 3 University of Toronto Faculty of Medicine, Medical Sciences Building, 1 King’s College Circle, Toronto, ON M5S 1A8 Canada

**Keywords:** End-of-life communication, Death and dying, Assisted living, Palliative care, Advance directive, Nursing homes

## Abstract

**Objectives:**

Despite known benefits, advance care planning (ACP) is rarely a component of usual practice in long-term care (LTC). A series of tools and workbooks have been developed to support ACP uptake amongst the generable population. Yet, their potential for improving ACP uptake in LTC has yet to be examined. This study explored if available ACP tools are acceptable for use in LTC by (a) eliciting staff views on the content and format that would support ACP tool usability in LTC (b) examining if publicly available ACP tools include content identified as relevant by LTC home staff. Ultimately this study aimed to identify the potential for existing ACP tools to improve ACP engagement in LTC.

**Methods:**

A combination of focus group deliberations with LTC home staff (*N* = 32) and content analysis of publicly available ACP tools (*N* = 32) were used to meet the study aims.

**Results:**

Focus group deliberations suggested that publicly available ACP tools may be acceptable for use in LTC if the tools include psychosocial elements and paper-based versions exist. Content analysis of available paper-based tools revealed that only a handful of ACP tools (32/611, 5%) include psychosocial content, with most encouraging psychosocially-oriented reflections (30/32, 84%), and far fewer providing direction around other elements of ACP such as communicating psychosocial preferences (14/32, 44%) or transforming preferences into a documented plan (7/32, 22%).

**Conclusions:**

ACP tools that include psychosocial content may improve ACP uptake in LTC because they elicit future care issues considered pertinent and can be supported by a range of clinical and non-clinical staff. To increase usability and engagement ACP tools may require infusion of scenarios pertinent to frail older persons, and a better balance between psychosocial content that elicits reflections and psychosocial content that supports communication.

## Background

Long-Term-Care (LTC) homes are a major site of death for older adults globally [1, 2]. Yet, many LTC facilities still lack formalized palliative care programs, resulting in sub-optimal end of life care [3, 4].

Advance care planning (ACP) is an important component of a comprehensive palliative approach program because it empowers individuals with non-reversible health conditions to reflect on, communicate, and sometimes document their values, beliefs, and preferences for future end of life care [5, 6]. As such, ACP provides a mechanism to ensure that persons with life-limiting conditions remain at the centre of their own care by providing avenues for communicating care preferences to family/close friends, legally appointed decision-makers and health providers, when capacity for reflection, communication, and decision-making is consistently present [7–9].

When ACP is introduced in LTC, it is associated with reductions in hospital admissions, increased concordance between preferred and received care, and decreases in stress, depression, and anxiety for residents and their families [5, 10–12].

Despite the known benefits, ACP is rarely a component of usual practice in LTC [13, 14]. Key barriers to ACP engagement include lack of resident and family preparedness [15, 16], limited staff time, and discomfort and uncertainties from all parties regarding what to discuss beyond single medical decisions and funeral planning [17–20]. ACP programs that include multiple steps have shown some successful outcomes in increasing ACP engagement in LTC [13]. Such programs typically offer direction to clinicians around making people aware of ACP (e.g. how to explain what ACP is, what it involves and why it is important), facilitating ACP conversations (e.g. questions to ask to elicit personal preferences for care, values and wishes that may guide future decisions) and mechanisms to help people identify what to communicate to others such as family or appointed decision-makers [21]. However, not only are such programs difficult to sustain in daily practice without leadership support and/or dedicated staff time [13, 21], many are designed to be implemented by clinical staff such as nurses and physicians, whose presence in LTC is limited [16]. For example, in the context of LTC between 70 and 90% of the hands-on care is provided by non-clinical staff such as nursing aides and dietary aides [22, 23]. Hence, ACP materials that do not heavily rely on clinical staff for facilitation may go a long way in improving ACP uptake in LTC [24].

Interactive tools like workbooks, videos, and card games designed for self-use have recently been developed to encourage the general population to raise awareness regarding the importance of ACP and encourage reflection and communication with family, friends, and health providers [25, 26]. Given the self-directed nature of these materials, these tools may be suited for distribution and use with both non-clinical staff and clinical staff in LTC [27]. Yet, LTC home staff’s perceptions regarding the acceptability and usability of these materials has not been explored.

Redressing this gap in the literature, this study combined focus groups discussions with LTC staff and a content analysis of available ACP tools to answer the following research questions: (a) what content and formats may make ACP tools acceptable and usable in LTC from the perspective of staff, and (b) how, if at all, does the content and format of available ACP tools align with the recommendations and preferences of LTC home staff? Ultimately, this study was meant to be serve as a first step towards improving ACP uptake in LTC by identifying the types of ACP tools staff thought may support ACP reflections and conversations in LTC.

## Methods

This study employed a qualitative sequential exploratory design to meet its aims in two steps [28]. To address the first research question, focus groups were held with LTC home staff to elicit views on the content and format thought to support ACP tool usability in LTC. To address the second research question, a content analysis of publicly available ACP tools was conducted to explore whether available ACP tools included the content and formats identified as important to support usability by LTC staff in step one.

The research was conducted in accordance with the standards of the Tri-Council Policy Statement for Ethical Conduct for Research Involving Humans 1998 (with 2000, 2002, and 2005 amendments). Procedures were approved by the Office of Research Ethics Boards at McGill University and McMaster University.

### Step one: focus groups

#### Focus group recruitment and data collection

Staff from four LTC homes, known by the research team to be palliative leaders in their respective homes, were invited to participate in focus group deliberations. The four homes within which staff were recruited were partners in a larger study aimed at strengthening a palliative approach in LTC [21]*.*

Recruitment took place 1 month prior to each focus group and was limited to staff who had been active members of palliative champion teams (*n* = 55). All potential participants were contacted directly by the research team via email or verbal invitation.

Focus groups lasted approximately 60 min and were facilitated by two members of the research team. Participants were first provided with a range of ACP tools including one card game [29], two workbooks: one which culminated in the development of advance directives [30] (Five Wishes- https://fivewishes.org) and another which did not [31] (Conversation Starter Kit- https://theconversationproject.org/starter-kits), and an interactive website [32] (Conversations Matter- http://goals.conversationsmatter.ca.s3-website-us-east-1.amazonaws.com). In the first 20 min the facilitators briefly presented each tool, encouraging participants to scan the material and jot down thoughts and reactions (5 min each). In the following 40 min participants were invited to share reactions to the tools and discuss their thoughts on using them in a LTC environment. Written informed consent was attained on the day of focus group deliberations.

#### Data analysis focus groups

All focus group deliberations were audio-recorded and transcribed, and field notes were taken throughout. Transcripts and field notes were analyzed by two members of the research team in two steps guided by the principles of content analysis [33]. In step one, researchers independently and then together coded comments about each tool as either strengths or limitations. In step two, commonalities and discrepancies between tools and across groups were examined to identify if particular tools were consistently viewed either positively or negatively and to illuminate overall themes related to the strengths and limitations of all tools to support ACP discussions in LTC.

### Step two: content analysis search strategy

Informed by findings from our focus group deliberations, we aimed to identify and analyze publicly available paper-based ACP tools with psychosocial content that could be easily accessed and utilized by residents, families, and staff in LTC. We sought to identify tools through a grey literature search using Google as our search engine. The key words “Advance Care Planning Tools” were used to locate paper-based materials. We also solicited key informants for additional tool identification.

Guided by the themes emerging from our focus group deliberations, tools were excluded from review if they (a) focused exclusively on recording medically-oriented advance directives or decisions including do-not-resuscitate orders or the cessation of particular treatments such as kidney dialysis for advanced renal failure; (b) were not available in printable form; (c) were designed for use only by clinically trained staff and (d) lacked information on ACP and direction to guide engagement and use. The research team also decided to exclude tools that were (e) developed for substitute decision-makers only because we were interested in materials that would support the inclusion of residents in their own future care planning [34, 35].

Two researchers were involved in determining tool retention/exclusion. The first researcher conducted an initial review of identified tools once exclusion criteria were established. The second was consulted with a random selection of tools slated for exclusion and purposefully when uncertainties arose.

### Content analysis ACP tools

The content of the workbooks was analyzed in three stages by the two researchers responsible for tool selection [36]. In the first stage we used conventional content analysis to code excerpts from each guide into the broad ACP areas of reflection (components designed to encourage thinking about personal values, goals, and preferences), communication (components designed to encourage conversations between patients, decision-makers, and others, including steps to take to begin the conversation), and documentation (components left for patients to record any wishes or preferences that could be referred to by others). These aspects are commonly denoted in the literature as critical and unique steps in the ACP process [9, 37, 38]. Informed by the findings from our focus group deliberations, only psychosocially-oriented excerpts were analyzed. An excerpt was considered psychosocial if it referred to a value (e.g. autonomy, independence, quality of life) or a care preference that was social (e.g. wish for family support or involvement); emotional (e.g. peace of mind); environmental (e.g. private room, location of death) or spiritual/religious (e.g. last rites; preferred music).

In the second stage, through discussion and a collective review of coded excerpts, the team further refined the categories for reflection, communication, and documentation into what we considered vague psychosocial content (e.g. reflecting on what one considered quality of life) and specific psychosocial content (e.g. reflecting on music preferences at end of life). We also specified whether psychosocial reflections, communication, and documentation were geared towards future end of life care or about designating a decision-maker, as both are considered unique aspects of ACP [9, 37, 38]. Noting the extent to which substitute decision-makers were encouraged to participate in reflections, communication, and documentation seemed important both because these distinctions were emerging from the content and because recent models of ACP recognize that decision-maker involvement in all stages of the ACP process can encourage ACP activation for older adults living with frailty [39, 40].

In the third and final stage, the frequency with which each tool had psychosocial content falling within each category was recorded to illustrate the most and least common applications of psychosocial content. At this stage, two researchers independently coded content. Any identified discrepancies were discussed with a third researcher who helped make a final determination based on consensus. We also created formatting-related categories noted in the literature and our focus group deliberations to support readability for older persons, including tool length (discussed in all four focus groups), use of pictures/images, and inclusion of scenarios applicable to older persons [41].

## Results

### Focus group results

Out of the 55 invited staff, 32 participated in four focus groups, including 23 clinical staff (13 nurses, seven allied health professionals and three clinical leaders) and nine non-clinical staff (four support staff such as dietary and recreational aides and five trainees/volunteers).

While staff across groups expressed divergence and uncertainty around *when* and *by whom* ACP discussions should be initiated in LTC, many suggested that with the right content and format ACP tools could provide useful guidance for staff, families, and residents. The sections that follow provide an overview of the content and format staff felt would contribute to ACP tool usability within a LTC home environment.

#### Tools with ACP information and direction are key

ACP tools that provided information on the importance of ACP and the role of decision-makers, gave examples and scenarios that could help stimulate reflection, and included a range of prompting questions to guide discussions were seen to be particularly helpful to guide ACP discussions between staff, residents, and families. Participants felt *“[tools that] create an incentive [by including information] outlining what might happen if you don’t have an advance care plan …*. *[would] make them aware of the importance*” (Focus Group 3). Others suggested that tools that offered “*good ways to start a conversation*” (Focus Group 2) and “*specific examples and prompts*” (Focus Group 4) were useful as they provided staff with the support they needed to activate such conversations and offered the focus and direction required to elicit useful reflection and dialogue. Prompting residents to list the top three things they want their families/friends to know about their end of life preferences or asking them if they would be okay with spending their last days in hospital were provided as examples of prompts and questions with the right balance between specificity and openness.

#### Tools with a psychosocial focus are useful

Focus group deliberations further suggested that while tools with a strong or sole medical focus may “*fit well with charting requirements*” (Focus Group 3) they were limited in their usefulness because they needed to be completed with physicians and nurses, and failed to include topics of critical importance to guide care in LTC. As one participant stated “*I don’t want to die by myself, I don’t want to die alone, I want to be at the home, I don’t want to be a burden on my family … those are the key issues we hear often in LTC*” (Focus Group 1). Overall, participants within and between groups suggested that “*having psychosocial aspects covered in a tool”* (Focus Group 4) alongside medical concerns and issues could be particularly useful because it expanded the scope of professionals who could support implementation and covered topics of critical importance to residents and families in LTC. Psychosocial topics considered of high relevance in a LTC environment included views on family involvement, beliefs about quality of life/death, environmental conditions thought to enhance care, and religious/spiritual preferences.

#### Divergent tool options, paper-based formats, and moderate tool length may support usability in LTC

Most focus group participants suggested that no one tool would ever be appropriate for all LTC residents. Rather, participants expressed the desire for blending tools or having a few tools to refer to. As one participant stated, “*You need to see the needs of a particular [resident or] family and choose one out of two or three favourite resources”* (Focus Group 4).

Although opinions varied regarding which tool of those reviewed was most useful for a LTC environment, two format issues were consistent within and across groups: *“Hard copies have to be available”* (Focus Group 1) so that computer accessibility and literacy are not an obstacle, and tools that are too long will be overwhelming for residents, families and staff (mentioned in all focus groups).

### Content analysis results

A total of 611 tools were identified through our key word Google search, of which 489 (80% of identified tools) were excluded because of their sole medical focus. An additional 49 tools (8%) were excluded because they were not paper-based and/or required internet access to be utilized, and 32 tools (5%) could not be accessed because the website housing them had expired or the tool was otherwise unavailable. A further 12 tools (2%) were excluded because they were designed solely for clinicians or decision-makers.

Hence, we retained 29 ACP workbooks for further review. To ensure we had located all viable paper-based ACP materials, we emailed the list of 29 tools to our broad network of national and international collaborators and researchers engaged in the areas of palliative care, long-term care, and aging (*N* = 75). This process yielded an additional 3 tools. Table 1 provides a brief overview of all 32 tools retained for analysis including where they can be located and whether they have been evaluated in the scholarly literature. It is noteworthy that of the 32 tools located only three appear to have been formally evaluated as evidenced by a search in the published literature and email-outreach to all organizations wherein tools were housed. Table 2 reports results of our content review. These findings are also reported below.

**Table 1:** Overview of Tools Reviewed

Tool Name	Description	Studies published on the piloting and evaluation of the tool	URL
1. Five Wishes Advance Directive	Living will that allows users to select an SDM and easily organize their psychosocial EOL care preferences in conjunction with their medical treatment preferences, by selecting items from pre-populated lists.	Chovan, 2007; Wiener et al., 2008	https://fivewishes.org/shop/order/product/five-wishes-advance-directive
2. Respecting Patient Choices: Information Booklet, Planning Guide, and Advanced Care Plan (Aged Care)	The Information Booklet contains general guidance on advanced care planning using clinical vignettes and FAQ-style questions. The Planning Guide offers space to write reflections on quality of life as well as medical treatment. The Advanced Care Plan (Aged Care) is a formalized directive which prompts the user to record general values, EOL care goals, and specific psychosocial and medical care preferences.	Detering et al., 2014; Seal, 2007, Silvester et al., 2013;	Tool provided via network of research collaborators.
3. Graphic Values History Tool	This tool is highly visual and accessible as it illustrates each reflective prompt by an accompanying symbol or graphic. It is divided into 5 different sections, guiding users to consider themes related to quality of life, value ‘tradeoffs’, considering whether certain health conditions are worse than death, the impact of their decisions on others, and their religious/spiritual/cultural beliefs.		Tool provided via network of research collaborators.
4. Speak Up: Advance Care Planning Workbook	Workbook which carefully defines ACP and emphasizes its importance, while providing reflective prompts, space to indicate one’s substitute decision-maker (SDM), and opportunities to write down thoughts and wishes about EOL care.		https://www.chpca.ca/product/advance-care-planning-workbook-national-edition-not-for-ontario-residents/
5. Your Life Your Choices	Workbook that uses case examples, legal and medical information, and thought-provoking questions and interactive written exercises to encourage users to think about and communicate their EOL preferences, while emphasizing the importance of ultimately documenting them.		https://www.elderguru.com/downloads/your_life_your_choices_advance_directives.pdf
6. American Bar Association: Tool Kit for Health Care Advance Planning	This “Kit” is composed of 10 different tools, purposefully structured to guide users through selecting an SDM, considering their values, indicating and communicating EOL care preferences, and ensuring decision-makers understand these choices via a “quiz”.		https://www.americanbar.org/groups/law_aging/resources/health_care_decision_making/consumer_s_toolkit_for_health_care_advance_planning/
7. Finding Your Way: Medical Decisions When They Count Most	Informational booklet that uses case vignettes, medical and general information, and reflective questions to prompt the user to consider what they value in terms of their life and end-of-life treatment, culminating by encouraging the creation of advance directives.		https://coalitionccc.org/wp-content/uploads/2014/02/Finding-Your-Way-English.pdf
8. Your Conversation Starter Kit	Workbook that provides users with interactive prompts regarding their personal and life values, and carefully guiding them through communicating their EOL preferences.	Lum et al., 2016	https://theconversationproject.org/wp-content/uploads/2017/02/ConversationProject-ConvoStarterKit-English.pdf
9. Alberta Health Services: Conversations Matter	Booklet designed to get users thinking and learning about their own health circumstances, and considering and communicating their EOL care preferences, via case vignettes, information about advance care planning, and guidance about documentation in directives.		https://myhealth.alberta.ca/Alberta/AlbertaDocuments/conversations-matter-guide-english.pdf
10. Dying with Dignity: Advance Care Planning Kits	Logically ordered information piece, specific to provincial jurisdiction, that takes users through understanding personal psychosocial directives, considering personal values, considering medical priorities, why an SDM should be named, talking to this person, and finally recording advance directives and SDM designations.		https://www.dyingwithdignity.ca/download_your_advance_care_planning_kit
11. Dying Matters: Resources	Series of eye-catching leaflets on various EOL topics. A sample of 13 were reviewed which emphasized the importance of advance care planning and promoted conversations on the subject, using examples, checklists, information and guidance.		https://www.dyingmatters.org/overview/resources
12. My Voice: Advance Care Planning Guide	Workbook that provides information about ACP, illustrated by case vignettes, and culminates in an opportunity for users to record general beliefs, values, and wishes, and to complete legally binding representation forms and an advance directive.		https://www.health.gov.bc.ca/library/publications/year/2013/MyVoice-AdvanceCarePlanningGuide.pdf
13. The Critical Conditions Planning Guide	Workbook that uses case vignettes and exercises to encourage users to discuss their EOL wishes, and to reflect on and record their life values and preferences, ultimately guiding them in the completion of an advance directive.		https://www.hcethics.org/docs.ashx?id=574714
14. Acclaim Health	Document that provides guidance and information about EOL care planning while emphasizing its importance, and encouraging users to identify and communicate their preferences and values.		Tool provided via network of research collaborators.
15. Begin The Conversation	Purposefully structured 7-step workbook that encourages patients to reflect on, communicate and formally record their medical and psychosocial preferences for EOL care, by presenting information and statistics about the importance of advance care planning, and reflective exercises.		http://www.begintheconversation.org/begin/act/
16. Compassion and Choices: My End-of-Life Decisions – An Advance Planning Guide and Toolkit	Workbook that provides information about decision-making and directives, includes values exercises, and emphasizes communication, in order for users to ultimately complete several advance directive documents.		https://compassionandchoices.org/wp-content/uploads/My-End-of-Life-Decisions-Guide-Online-Interactive-Version-FINAL-7.1.20.pdf
17. Deathwise: End-of-Life Binder Worksheets	Tool that guides users to consider and communicate their values, and that offers opportunity to record various important personal information including healthcare wishes, memorial service and obituary preferences, contact information of loved ones, and more.		http://deathwise.wpengine.com/wp-content/uploads/2013/08/End-of-Life-Binder-Worksheets.pdf
18. Stanford Letter Project: What Matters Most	Short letter template for patients to send to their physician, outlining key values and EOL care preferences, as intended to be kept in their medical records.		http://med.stanford.edu/content/dam/sm/letter/documents/Letter-English.pdf
19. Speak Up Ontario: Thinking about my wishes for future health care	Short workbook that uses interactive exercises to prompt users to think about their values, wishes and preferences for EOL, with a medical focus. It emphasizes the importance of communicating these thoughts with one’s SDM and others.		https://www.speakupontario.ca/wp-content/uploads/2018/07/ACP-Patient-Template-En-SpeakUp-Ontario.pdf
20. Winnipeg Regional Health Authority: Advance Care Planning	Workbook encouraging care planning by providing information and prompting users to reflect on and communicate their values and beliefs, and to ultimately complete an enclosed advance directive.		https://professionals.wrha.mb.ca/files/acp-workbook.pdf
21. Nova Scotia Health Authority: Advance Care Planning Patient & Family Guide	Workbook that provides extensive information about medical treatments, decision-making, and directives, and guides users through values exercises, culminating in the creation of a directive including both psychosocial and medical preferences.		http://www.nshealth.ca/sites/nshealth.ca/files/patientinformation/1942.pdf
22. Honoring Choices Minnesota: Choose Your Agent, Sample Values Statements, Health Care Directive, and Guide to Completing Your Directive	Series of information packages that prompt the patient to choose an SDM, think about their values for EOL care, and use those values to complete an advance directive including both medical and psychosocial components, with extensive guidance.		https://www.honoringchoices.org/tools-resources/how-to-start
23. Advance Care Planning: A Catholic, Faith-Based Perspective	Information booklet that provides users with guidance about making EOL decisions in keeping with the teachings of the Catholic Church. It encourages reflection and communication, and offers opportunity to record a directive involving psychosocial as well as medical aspects of care.		https://www.chabc.bc.ca/wp-content/uploads/2017/05/ACP-document.pdf
24. Baycrest: Advance Care Planning-Making Your Health Wishes Known	Tool that provides important guidance about EOL care planning and decision-making, while using vignettes and information to subsequently prompt the patient to think about, speak about, and record what is important to them.		https://www.baycrest.org/Baycrest_Centre/media/content/form_files/ACP_MAKING-WISHES-KNOWN.pdf
25. The College of Family Physicians of Canada: Advance Care Planning Resource for Patients	Short document directed at patients that explains advanced care planning and what the role of an SDM is. It urges communication with family, friends, and medical professionals, prompts users to start thinking about their psychosocial and medical wishes for the end of their life, and recommends documentation.		https://portal.cfpc.ca/resourcesdocs/uploadedFiles/Resources/Resource_Items/Patients/AdvanceCarePlanning_ENG-Final.pdf
26. Northern Ireland Palliative Care Tools & Guidance: Advance Care Planning - Your life and your choices: plan ahead, Your Checklist for Planning Ahead, My Advance Decision to Refuse Treatment (ADRT), and Record my Wishes	Collection that provides users with extensive guidance about why, how, and with whom to plan ahead, through information, vignettes, and exercises, culminating in the opportunity to record wishes as reflections as well as in legally binding documents.		http://www.professionalpalliativehub.com/guidelines/northern-ireland-palliative-care-tools-guidance/advanced-care-planning
27. Michael Garron Hospital: Advance Care Planning Workbook	Workbook that defines and promotes advanced care planning and encourages users to learn about their health, choose an SDM, think about their values and wishes via a writing exercise, and share their wishes with family and healthcare providers.		https://www.tehn.ca/sites/default/files/file-browser/acp_workbook_mgh_final_feb_2016.pdf
28. Perth and Smith Falls District Hospital: Advance Care Planning in Ontario	Step-by-step workbook that provides information and reflective questions to prompt users to think about their preferences, learn about their health, choose an SDM, while highlighting the importance of ultimately sharing and recording their wishes.		https://psfdh.on.ca/wp-content/uploads/2017/09/CAPCE-Final-Project-October-6-2017Advance-Care-Planning.pdf
29. Healthy New Hampshire: Advance Care Planning Guide	Guidebook that provides reflective questions and extensive information about advance care planning, encouraging the careful selection of an SDM via an interactive exercise, and culminates in the completion of an advance medical directive.		https://www.healthynh.com/images/PDFfiles/advance-directives/2017_ACPG_Final.pdf
30. Making Choices Michigan: Advance Directive- Durable Power of Attorney for Healthcare (Patient Advocate Designation)	Advance directive that focuses on documentation including the appointment of an SDM and the recording of both medical and psychosocial care preferences. It urges the importance of communicating with the selected SDM.		https://makingchoicesmichigan.org/wp-content/uploads/2018/08/MCM-AdvanceDirective-051817-fillable.pdf
31. Got Plans? Your Advance Care Planning Guide	Simple guidebook that prompts and describes the selection of an SDM, encourages reflection and communication via thought-provoking questions, and guides the user to go on to document wishes in an advance directive.		http://compassionatecarenc.org/wp-content/uploads/2017/04/GotPlansGuide_031017.pdf
32. Utah Commission on Aging: Tool Kit for Advance Healthcare Planning	Series of 10 tools that are systematically laid out, using exercises, information and advice, to prompt users to select their SDM, consider and communicate their medical and psychosocial wishes, and ensure their SDM understands, via an ‘IQ Test’. It culminates in an advance medical directive.		https://ucoa.utah.edu/_resources/documents/directives/tool-kit-2012.pdf

**Table 2:** Content Analysis of Retained ACP Tools (*N* = 32)

Domain		Frequency	Example
Psychosocial reflections	General	30 (94%)	“Think about what’s important to you and how your values help you make healthcare decisions”. (Michael Garron Hospital: My Health, My Wishes ACP Workbook)
	Specific	22 (69%)	“What cultural or traditional practices are important to you? Do you wish the plans to observe certain religious or non-religious beliefs?” (College of Family Physicians: ACP Resource for Patients)
Communication about psychosocial issues	General	32 (100%)	“To do: … discuss your thoughts with those close to you; your family, your GP and other involved health care providers.” (Respecting Patients’ Choices Planning Guide)
	Specific	5 (17%)	“{T} ell your SDM(s), family and friends: What is important to you at the end of your life; Religious readings or ceremonies you want to have; Music you want to listen to; Books you want to read or have read to you; Where you might want to spend the last days of your life.” (Acclaim Health)
Documentation of psychosocial directives	–	7 (22%)	“Goals for end-of-life care: What do you hope for most when you are near the end of your life? (For example: presence of family or other persons; access to places or items of significance; music; any personal, religious or cultural practices to be followed): {*lined writing space is provided*}”. (Respecting Patient Choices: Advance Care Plan (Aged Care))

### Readability and formatting

Retained tools ranged in length from 3 to 102 pages. Although a small minority were less than 12 pages (6/32, 19%), one third (11/32, 34%) exceeded 25 pages. Most tools in our review (21/32, 67%) included photos or graphics and offered vignettes or case situations to encourage ACP reflection (18/32, 56%). Workbooks often featured photos that appeared to be of people having discussions with loved ones, or graphics or symbols depicting certain healthcare scenarios such as hospitalization. Half of the tools reviewed included vignettes about the care of an older person (16/32, 50%), such as imagining oneself having Alzheimer’s disease and being unable to recognize loved ones or communicate with them, or of someone contracting pneumonia and moving from a nursing home to a hospital.

### Psychosocial content in tools

#### Psychosocial reflections

The vast majority of tools included in the review encouraged some form of psychosocial reflection (30/32, 94%). Common psychosocial reflections included asking users to think about or write down what constitutes quality of life, a good death, and/or what gives life purpose or meaning, through questions like *“What are your values and beliefs about death and dying? … What does suffering mean and what makes life worth living?”* [42]. These general reflections were framed as a way of beginning to uncover underlying beliefs, values, and preferences that could help to inform future decision-making. Some tools went a step further, moving from more global reflections to encouraging specific reflections about EOL preferences. Specific reflection about location of death was most common, as featured in 27/32 (84%) of tools, via prompts such as “*If possible, would I prefer to die at home, in a hospice or in the hospital? What might change my mind about my choice?”* [43]. Slightly fewer (22/32, 69%) included additional specific reflections, including what they think may bring them comfort in their final days, and whom, if anyone, they would elect to have by their side should their health deteriorate. Tools often provided reflective questions like “*If you could plan it today, what would the last day of your life be like? … What would you be doing? Who would be with you? What would you eat, if you were able to eat?...”* [44], or enabled users to select from listed items such as “*When I am nearing the end of my life I want: my family nearby; someone holding my hand; my religious leader to visit me …”* [45].

#### Communication about psychosocial issues

All tools (32/32, 100%) mentioned the importance of communicating and sharing reflections with families, friends, healthcare professionals, and substitute decision-makers, and many (27/32, 84%) encouraged users to think about who they may select as a substitute decision-maker in light of their reflections and preferences. Yet far fewer tools (14/32, 44%) provided users with tips on how to initiate conversations with families, friends, and decision-makers, how to speak with their decision-makers about their comfort acting in the role in light of their wishes (13/32, 41%), or provided guidance directly to substitute decision-makers regarding what they may ask or think about (7/32, 22%).

When communication tips were provided they tended to be general in nature, such as encouraging people to find the right time to have a conversation or providing them with an opening prompt such as *“I need to think about the future. Will you help me?”* [31]. Only a handful of workbooks (5/32, 17%) specifically encouraged the user to speak about their psychosocial preferences for end of life care with those close to them, via prompts including “*… ACP Conversations are a process so you do not have to think about this until you are ready. But if you have thought about it, tell your SDM(s), family and friends: what is important to you at the end of your life … music you want to listen to, books you want to read or have read to you …”* [46] or “*What is important [in the following exercise] is that you understand what each person involved in your conversation wants for himself or herself … Would you like someone to be with you when you die? Who?”* [47].

#### Documentation of psychosocial directives

While documentation is a common element of medically-oriented ACP workbooks, only a fifth (7/32, 22%) of the workbooks we reviewed included a formal opportunity to document psychosocial preferences for EOL. Those that included a documentation component encouraged users to turn their reflections into a formalized plan that identified specific psychosocial directives such as “*I wish to have religious readings and well-loved poems read aloud when I am near death”* (Five Wishes); “*I want X at my bedside [at end of life]”: … [I] want someone to hold [my] hand”* [48]. While not necessarily legally binding, this process ensures the translation of reflections and discussions into actionable conditions that users hoped would be applied in their final moments of life.

## Discussion

This study used the combination of focus group deliberations and content analysis of existing ACP tools to explore the types of ACP tools LTC home staff would consider using to improve ACP uptake in LTC. Our focus group deliberations suggested that ACP tools that include information about the importance of ACP and interactive exercises and questions that stimulate targeted reflection may improve ACP engagement in LTC provided the materials are available in paper-based formats. Focus group deliberations further uncovered that tools with limited psychosocial content were ill-suited for LTC. Yet our content review identified that a striking 80% (489/611) of existing ACP tools focused solely on supporting discussions and documentation of preferred *medical care* at EOL including non-resuscitation and non-intubation orders (i.e. advance directives). Current ACP research in LTC affirms the high prevalence of medically-focused ACP materials, as most studies exploring ACP impacts in LTC actually look more specifically at the implementation of advance medical directives [49]. While outcomes from this work have certainly shown some promise, our findings suggest such interventions are unlikely to be successfully adopted into usual practice in LTC if psychosocial issues are excluded, such as preferences for family involvement/non-involvement in EOL care, views about dying alone, spiritual beliefs that may provide comfort at EOL, and values related to quality of life/quality of care [34, 50].

Quite possibly the recurrent distribution and testing of medically-oriented ACP materials also perpetuates the limited understanding of ACP amongst residents and families in LTC reported in the literature [51]. Until such time as ACP is understood more broadly to include reflections on values, beliefs, and preferences that can be used to inform in-the-moment decisions rather than solely on the identification of pre-specified medical decisions that may not be easily implemented within unforeseen contexts, it will not be consistently implemented in LTC or elsewhere [8, 38]. It is noteworthy that when encouraged to think broadly about ACP, residents and families in LTC affirm the importance of discussing resident’s values and wishes for physical, social, spiritual and psychological care [52]. It is also noteworthy that such discussions are considered best initiated by staff who know the resident well, as this can create the comfort necessary to discuss emotionally difficult topics such as future end of life care [52].

Our initial screening of available ACP materials resulted in the retention of 32 tools found to include some psychosocially-oriented content. The vast majority of these tools encouraged reflections about psychosocial issues pertinent for EOL decision-making in LTC such as beliefs about quality of life/death. Many also went a step further to help users unpack such reflections by orienting them towards smaller, more specified reflections such as preferred location of death, rituals of importance, and specific actions that may provide comfort. Encouraging specific reflections that can be more easily communicated has been found to be a useful approach to ACP activation [9]. It was also identified as important by LTC staff in the current study.

However, most of the tools we reviewed included less content and direction around how to communicate psychosocial preferences to decision-makers, family/friends, and health providers and even fewer offered direction on how to select decision-makers and speak to them about their roles and responsibilities. Further, almost none of the tools offered formalized directions on how to transform wishes and preferences into a documented plan that included psychosocial elements. While some have argued that pre-prescribed plans are of limited use because they fail to account for the specific and contextualized decisions that emerge at EOL [53], selection of and communication with substitute decisions-makers and health providers about roles, responsibilities, decisional flexibility, and desired decisional involvement has been increasingly recognized as a critical component of ACP [8, 9, 27, 38, 54] as these discussions provide direction to legally-appointed decision makers regarding issues of high importance such as residents’ expectations for shared decision-making despite diminished capacity [55]. Although workbooks alone may not be sufficient to support the movement from reflection to oral or written communication, more orientation towards this challenging aspect of ACP could provide residents, families, and staff at all levels with tips and directions that may encourage communication on this emotionally complex and difficult topic.

In terms of applicability to a LTC home environment, two thirds of the tools reviewed were a moderate length (12–25 pages), most included graphics and images supporting readability, and half infused scenarios and examples relatable to circumstances/situations older persons living with frailty may face. Hence, some of the psychosocially-oriented workbooks and materials available may still require adaptations for a LTC environment.

### Implications and recommendations

Our findings provide some direction to improve ACP engagement and uptake in LTC. First, our review identified a series of tools that include psychosocial components. We encourage LTC home administrators and directors of care to use the list provided as a starting point to select material for possible distribution. We also encourage them to reconsider any tendencies to use materials with a strictly medical focus. Second, prior to implementation within a selected LTC home environment, we suggest preferred material be altered (if required) to include typical LTC home scenarios that may encourage ACP reflections and to ensure a relatively equal balance between prompting questions and exercises that encourage reflections and those that encourage communication and documentation. With the advent of the COVID-19 pandemic, additional adaptations or companion resources may be crucial to ensure the relevance of these tools. A number of the tools we identified have already evidenced this, including the Speak Up tool (https://www.speakupontario.ca/wp-content/uploads/2020/04/Engaging-in-Advance-Care-Planning-for-COVID-19.pdf). Third, while staff expertise in ACP tool selection represents a first step towards improving ACP uptake in LTC, implementation strategies that include discussions and planning around staff roles and expectations must follow. In keeping with preferences expressed by residents and families alongside staff ratios in LTC, strategies that specify a role for non-clinical staff such as care aides, activity aides or dietary aides should be considered [35, 52]. For example, non-clinical staff with a strong connection to a resident could either distribute the selected ACP tools or use them to guide questions and reflections within the context of usual care. If required, this process could be followed by a more structured care conference or bed-side check in led by nurses, social workers or physicians. Designating an ACP leader to plan for and oversee implementation could help to ensure that the strategies that are developed are adopted into practice [21].

Fourth, given the dearth of evidence located on the effectiveness or implementation of the identified tools we strongly encourage more research on ACP in LTC that evaluates the identified psychosocially-oriented ACP materials. At the time of writing, we were only able to locate published studies that empirically evaluated three of the tools we reviewed (see Table 1). While our review suggests these materials are of high relevance to a LTC environment, studies evaluating the distribution and use of such materials in LTC are warranted to maximize impact and uptake.

### Limitations

At the time of writing electronic materials were considered inaccessible for many residents in LTC and hence our review was limited to tools with paper-based options. In response to the COVID-19 pandemic and the need to ensure connections between residents and families, electronic communication has become more common in some LTC environments. It is possible that more psychosocially-oriented ACP tools are available in these digital formats and it may be timely to revisit the feasibility of electronically-based ACP materials in LTC.

While we conducted a systematic search to identify publicly available ACP tools, we did not perform a full systematic review on published work that may have evaluated these tools. Therefore we may have omitted some relevant evaluation studies on located ACP tools. However, our preliminary search suggested that the vast majority of the tools on our list have not been evaluated.

Our review failed to distinguish between workbooks that had some content applicable to a frail older population and those that were predominantly focused on issues related to aging and frailty. Hence our estimate of available psychosocially-oriented workbooks applicable to older persons in their current form may be inflated. We consider this a minor limitation as we expect all identified tools to require some adaptations to ensure applicability in local LTC environments.

Finally, although our focus groups included strong representation from key stakeholders implicated in the provision of care in LTC, non-clinical staff were underrepresented and no groups included the perceptions of physicians, who were invited but unable to attend. Future work would benefit from including physicians whose views may differ from that of other staff and ensuring more non-clinical staff representation whose voices in care provision in LTC are of critical importance.

## Conclusions

A handful of ACP tools currently exist that include psychosocial content of relevance to LTC. However, these tools may require infusion of scenarios pertinent to frail older persons, re-formatting, and a better balance between prompts encouraging reflections and those geared towards communication to improve usability and engagement by a range of LTC home staff.

## Data Availability

The datasets used and/or analysed during the current study are available from the corresponding author on reasonable request. A list and location of all ACP tools included in the content analysis (Phase II) is provided in the paper (see Table 1).

## Abbreviations

ACP: Advance care planning
LTC: Long-term care
